# Differential Sensitivity of Kupffer Cells and Hepatic Monocyte-Derived Macrophages to Bacterial Lipopolysaccharide

**DOI:** 10.31531/edwiser.jcegh.1000106

**Published:** 2019-09-03

**Authors:** Katherine Roth, Cheryl E. Rockwell, Bryan L. Copple

**Affiliations:** Department of Pharmacology and Toxicology and the Institute for Integrative Toxicology, Michigan State University, East Lansing, MI, USA

**Keywords:** Kupffer cell, Antibody, Macrophage, Lipopolysaccharide

## Abstract

The liver contains two distinct populations of macrophages, monocyte-derived macrophages (MDMs), which primarily reside proximal to the Glisson’s capsule and Kupffer cells, which reside within the sinusoids. Kupffer cells infiltrate the liver during embryogenesis and are replenished from local proliferation of mature Kupffer cells. By contrast MDMs arise from hematopoietic stem cells in the bone marrow and are replenishedfrom circulating monocytes. Studies have revealed that these two hepatic macrophage populations possess distinct transcriptomic profiles, suggesting that they may be functionally distinct. In the present study, we tested the hypothesis that MDMs and Kupffer cells are differentially sensitive to bacterial lipopolysaccharide (LPS). MDMs and Kupffer cells were purified to greater than 90% from the livers of mice by using magnetic beads labeled with Cx3cr1 antibody for MDMs and F4/80 antibody for Kupffer cells. Basal levels of tumor necrosis factor-α (TNF-α) mRNA were higher in MDMs when compared to Kupffer cells. After treatment with LPS, mRNA levels of TNF-α, Cxcll, and Cxcl2 were increased to a greater extent in MDMs when compared to Kupffer cells. To confirm these findings, Kupffer cells and MDMs were isolated from mice in which bone marrow transplantation was used to selectively tag cells arising from hematopoietic stem cells in adult mice. Similar to above, treatment of MDMs with LPS increased TNF-α, Cxcll, and Cxcl2 to a greater extent when compared to Kupffer cells. Collectively, these results indicate that MDMs exhibit a greater pro-inflammatory phenotype in the liver when exposed to LPS.

## Introduction

Hepatic macrophages arise from two distinct developmental lineages [1]. Kupffer cells, the resident tissue macrophages of the liver, develop from progenitor stem cells originating in the fetal yolk-sac [2,3]. These cells reside within the lumen of the hepatic sinusoids where they are positioned to detect and clear blood-borne pathogens, cellular debris, and other foreign material that may enter the portal circulation through the gut [4]. Kupffer cells are replenished by local proliferation of mature Kupffer cells under homeostatic conditions and after toxin-induced liver injury [5]. Under conditions of severe depletion, however, bone marrow-derived monocytes are recruited to the Kupffer cell niche where they ultimately mature into Kupffer cells [6,7]. In addition to Kupffer cells, a second, distinct population of hepatic macrophages exists under steady-state conditions that develops from monocytes arising from hematopoietic stem cells in the bone marrow [8]. These monocyte-derived macrophages (MDMs), also known as liver capsular macrophages, are positioned proximal to the Glisson’s capsule, a fibrous layer of connective tissue surrounding the liver [9,10]. They are also located around blood vessels within the liver [6]. Studies have revealed that MDMs protect the liver from pathogens that might invade from the peritoneal cavity by extending dendrites through the Glisson’s capsule and into the peritoneal cavity [8].

Recent studies have demonstrated that in addition to being ontogenetically and morphologically distinct, bone marrow-derived and yolk sac-derived resident hepatic macrophages possess distinct transcriptomic profiles [8,9,11]. While Kupffer cells displayed enhanced expression of phagocytosis-related genes, MDMs displayed enhanced expression of antigen-presenting genes [9]. However, beyond identification of transcriptional differences between the two populations, it remains to be demonstrated whether there are functional differences between these macrophage populations.

Kupffer cells and MDMs can be distinguished by expression of membrane markers. Whereas Kupffer cells are F4/80^hi^CD11b^low^Cx3Cr1^low^, monocyte-derived LCMs are F4/80^low^CD11b^high^Cx3Cr1^high^ under steady-state conditions [2,6]. In the present study, we exploited differences in these cell surface markers and used bone marrow transplantation to purify these two macrophage populations. Once purified, we determined the sensitivity of these two macrophage populations to bacterial lipopolysaccharide (LPS) by measuring proinflammatory cytokine expression.

## Materials and Methods

### Animals

6–12-week-old male C57Bl/6 mice (Jackson Laboratories, Bar Harbor, ME) were used for all studies. For bone marrow transplantation, Ptprc^a^Pepc^b^/BoyJ mice, a congenic C57Bl/6 strain, which expresses the CD45. 1 allele were transplanted into C57Bl/6 mice which express the CD45. 2 allele. Mice were housed in a 12 hours light/dark cycle under controlled temperature (18–21°C) and humidity. Food (Rodent Chow; Harlan-Teklad) and tap water were allowed ad libitum.

### Bone Marrow Transplant

Recipient mice (C57Bl/6, CD45.2) were irradiated with 11 Gy as a split dose (2 × 5.5 Gy, 3 hours apart) using a Cs^137^ irradiator (JL Shepherd, San Fernando, CA). For some studies, the midsection of the mouse was covered with 5 mm of lead to protect the liver from irradiation. After 18–24 hours, the irradiated C57B1/6 (CD45.2) mice were injected (retro-orbital injection) with 2 × 10^6^ bone marrow cells isolated from Ptprc^a^Pepc^b^/BoyJ (CD45.1) donor mice. MDMs and Kupffer cells were isolated 3–5 months after bone marrow transplant.

### Isolation of Kupffer cells and monocyte-derived macrophages from C57Bl/6 mice

To isolate Kupffer cells and MDMs, livers were perfused with collagenase (Collagenase H, Sigma-Aldrich) as described previously [12]. The hepatocytes were removed by centrifugation and the nonparenchymal cells were pelleted by centrifugation at 300 g for 10 minutes. 1 × 10^8^ nonparenchymal cells were resuspended in 60 μl of MACS Buffer (2.5 g bovine serum albumin, 0.416 g EDTA, and 500 mL PBS) containing 12 μl anti-CD11c antibody (Miltenyi Biotec, Bergisch Gladbach, Germany). The cell suspension was incubated for 10 minutes in the dark at 4°C and then washed with 10 mL of MACS buffer. The cells were centrifuged at 300 g for 10 minutes and the resulting pellet was resuspended with 500 μl MACS Buffer and applied to MACS LS columns (Miltenyi Biotec). The column was rinsed 3 times with 3 ml of MACS buffer. Unlabeled CD11c-negative cells passing through the column were collected and centrifuged at 300 g for 10 minutes. The pellet was resuspended in MACS Buffer containing anti-CX3CR1 antibody (Miltenyi Biotec) (12 μl CX3CR1 antibody in 60 μl MACS buffer per 10^8^ cells). The cell suspension was incubated for 10 minutes in the dark at 4°C and then washed with 10 mL MACS buffer. The cells were centrifuged at 300 g for 10 minutes. The cell pellet was resuspended and applied to MACS LS columns (Miltenyi Biotec). Monocyte derived CX3CR1-positive macrophages were collected by removing the column from the midiMACS Separator and rinsing the column with 5 ml MACS buffer. The CX3CR1-negative flow-through was collected and centrifuged at 300 g for 10 minutes. The pellet was resuspended in MACS Buffer with biotinylated anti-F4/80 antibody (Miltenyi Biotec). The cell suspension was incubated for 10 minutes at 4°C and then washed and centrifuged as above. Streptavidin microbeads (Miltenyi Biotec), diluted 1:10 in 60 μl of MACs buffer, were added to the cell pellet and incubated for 10 minutes at 4°C. The pellet was resuspended in 500 μl MACS Buffer and applied to MACS LS columns. Kupffer cells were collected by removing the column from the midiMACS Separator and rinsing the column with 5 ml of MACS buffer.

### Isolation of Kupffer cells and monocyte-derived macrophages from bone-marrow transplanted mice

Livers from bone marrow transplanted mice were perfused and digested with collagenase. Nonparenchymal cells were separated from hepatocytes by centrifugation. To isolate Kupffer cells, half of the nonparenchymal cell fraction was incubated with anti-CD45.2 antibody for 10 minutes at 4°C (Miltenyi Biotec), followed by incubation with Streptavidin beads for 15 minutes at 4°C. The cell suspension was then washed, resuspended in 500 μl MACS Buffer, and applied to mAcS LS columns. The CD45.2-positive flow-through cells were then incubated with biotinylated anti-F4/80 antibody for 10 minutes at 4°C, followed by incubation with Streptavidin beads for 15 minutes at 4°C. The cell suspension was then washed, resuspended in 500 μl MACS Buffer, and applied to MACS LS columns. Kupffer cells were collected by removing the column from the midiMACS Separator and rinsing the column with 5 ml of MACS buffer.

To isolate MDMs, the remaining half of the nonparenchymal cells were incubated with anti-CD45.1 antibody for 10 minutes at 4°C (Miltenyi Biotec), followed by incubation with Streptavidin beads for 15 minutes at 4°C. The cell suspension was washed, resuspended in 500 μl MACS Buffer, and applied to MACS LS columns. MDMs were collected by removing the column from the midiMACS Separator and rinsing the column with 5 ml of MACS buffer. The pellet was resuspended in MACS Buffer containing anti-CX3CR1 antibody (Miltenyi Biotec) (12 μl CX3CR1 antibody in 60 μl MACS buffer per 10^8^ cells), and the CX3CR1 positive MDMs were collected as described above.

### Flow cytometry

The Kupffer cells and MDMs isolated using magnetic bead separation were washed and resuspended in FACS buffer (PBS, 1% FBS). The cells were then incubated with Fc blocking buffer (BD Biosciences; diluted 1:20) for 10 mins at 4°C. The cells were then rinsed and centrifuged at 300 g for 5 minutes. The cell pellet was then resuspended with anti-F4/80 Alexa Fluor-488 and anti-CD11b APC/Cy7 (BioLegend, San Diego, CA) and incubated for 30 minutes at 4°C. The cells were then washed twice and fixed in formalin (Sigma) for 15 minutes. After cells were fixed, they were washed twice and resuspended in FACs buffer. Fluorescence was then detected using an Attune NxT flow cytometer (Life Technologies, Carlsbad, CA) and the data were analyzed using Attune NxT software.

### LPS treatment

Cells were seeded in 1 mL Dulbecco’s Modified Eagle Medium (DMEM) (Thermo-Fisher Scientific, Waltham, MA) supplemented with 10% FBS and penicillin/streptomycin in each well of a 12-well cell culture plate (Grenier Bio-One, Kremsmunster, Austria) for ~16 hours. The cells were then washed with PBS before adding 1 mL serum-free DMEM to each well. The cells were treated with 10 ng/ml lipopolysaccharide (LPS) (Sigma Chemical) for 3 hours at 37°C.

### Immunohistochemistry

Immunofluorescence was used to detect F4/80, CD45.1, and CD45.2 as described by us previously [13]. Briefly, frozen livers were cut into 8 μm sections, fixed in 4% formalin for 10 minutes, followed by blocking in 10% goat serum. The sections were then incubated with Alexa Fluor-488 anti-mouse CD45.1 antibody (Biolegend) diluted 1:100, Alexa Fluor-488 anti-mouse CD45.2 antibody (Biolegend) diluted 1:100, or rat anti-F4/80 antibody (Bio-Rad, Hercules, CA) diluted 1:500. The sections were then incubated with goat anti-rat secondary antibody conjugated to Alexa Fluor-594 diluted 1:500 (Thermo-Fisher Scientific).

### Real-Time PCR

RNA was isolated from cells by using the E.Z.N.A Total RNA Kit I (Omega Bio-Tek) according to manufacturer’s instructions. Real-time PCR was performed as described previously [12]. Real-time PCR was performed on a QuantStudio 7 Flex Real-Time PCR System (Thermo-Fisher) using the iTaq Universal SYBR green Supermix (Bio-Rad). The following primer sequences were used: TNF-α: Forward- 5’- AGGGTCTGGGCCATAGAACT-3’, Reverse- 5’- CCACCACGCTCTTCTGTCTAC-3’; Cxcll: Forward- 5’-TGGCTGGGATTCACCTCAAG-3’, Reverse- 5’- GTGGCTATGACTTCGGTTTGG-3’; Cxcl2: Forward- 5’ - CTCAGACAGCGAGGCACATC-3’, Reverse- 5’ -CCTCAACGGAAGAACCAAAGAG-3 ‘; Rpl13a: Forward- 5-GACCTCCTCCTTTCCCAGGC-3’, Reverse- 5’-AAGTACCTGCTTGGCCACAA-3’.

### Statistical Analysis

Results are presented as the mean + SEM. Data were analyzed by two-way Analysis of Variance (ANOVA) where appropriate. Comparisons among group means were made using the Student-Newman-Keuls test, with a criterion for significance of p < 0.05 for all studies.

## Results

### Purification of Kupffer cells and MDMs from mouse liver

**Figure 1:**
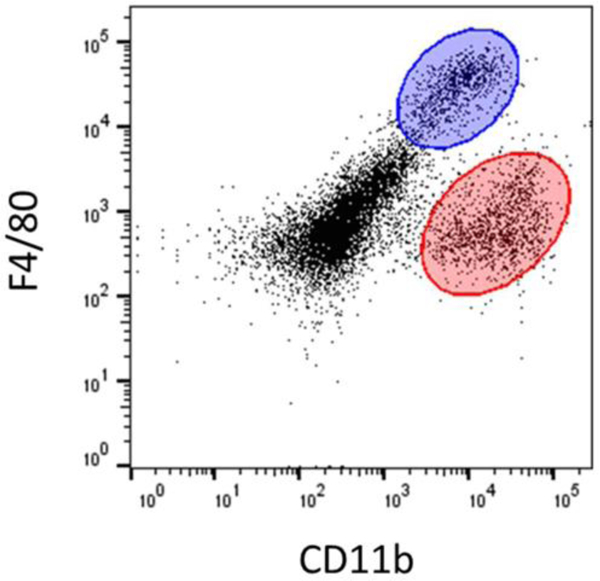
The nonparenchymal cell fraction was isolated from mice and flow cytometry was used to detect F4/80 and CD11b. The Kupffer cell population (F4/80^high^CD11b^low^) is highlighted in blue, while the MDM population (F4/80^low^CD11b^high^) is highlighted in red.

Flow cytometry was used to detect Kupffer cells (F4/80^high^CD11b^low^, blue oval) and MDMs (F4/80^low^CD11b^high^, red oval) in hepatic nonparenchymal cell fractions from untreated mice (Figure 1).

**Figure 2:**
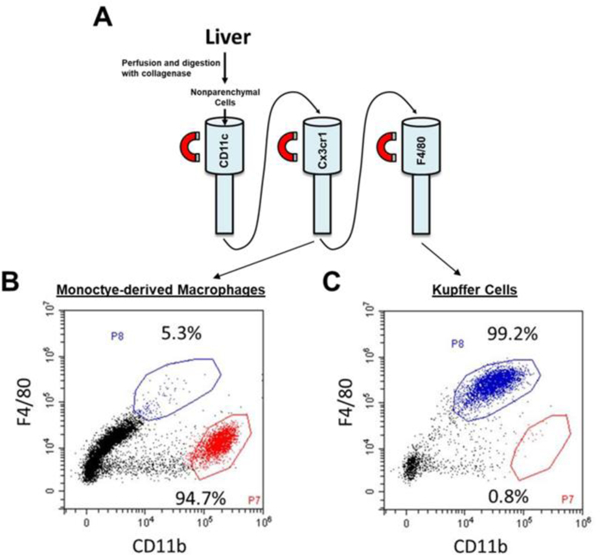
The nonparenchymal cell fraction was isolated from the liver. (A) Kupffer cells and monocyte-derived macrophages were purified by using immunomagnetic bead separation. Flow cytometry was used to detect F4/80 and CD11b in purified (B) MDMs (F4/80^low^CD11b^high^) and (C) Kupffer cells (F4/80^high^CD11b^low^).

**Figure 3:**
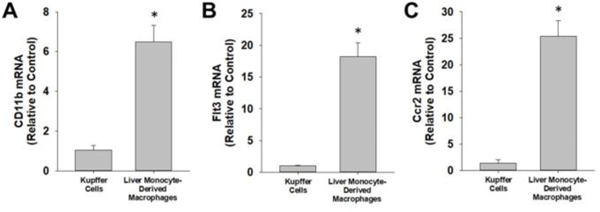
Kupffer cells and MDMs were isolated from the liver and mRNA levels of (A) CD11b, (B) Flt3, and (C) Ccr2 were measured by real-time PCR. Data are expressed as mean +/− SEM. [*Note:* *Significantly different from Kupffer cells at p<0.05].

Next, we used antibody-labeled magnetic beads to purify Kupffer cells and MDMs from nonparenchymal cell fractions. Antibody against CD11c, a protein expressed by dendritic cells, was first used to remove dendritic cells from the nonparenchymal cell fraction (Figure 2A) [14]. Next, antibody against CX3CR1 was used to purify MDMs and antibody against F4/80 was used to purify Kupffer cells (Figure 2A). Flow cytometry was then used to determine the purity of the Kupffer cell and MDM fractions. MDMs, identified as F4/80^low^CD11b^high^, were approximately 94.7% pure (Figure 2B), whereas Kupffer cells, identified as F4/80^high^CD11b^low^, were approximately 99.2% pure (Figure 2C). Further, basal expression of CD11b, Flt3, and Ccr2 mRNAs was greater in MDMs as reported previously (Figure 3).

### Differential upregulation of cytokines in Kupffer cells and MDMs by LPS

**Figure 4:**
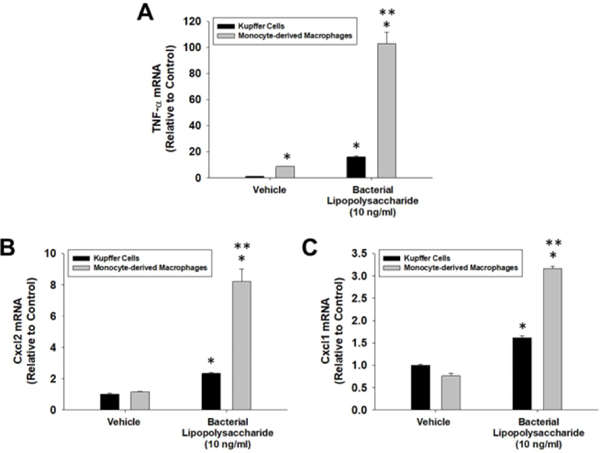
Kupffer cells and MDMs were isolated from the liver and treated with LPS or vehicle for 3 hours. mRNA levels of (A) TNF-α, (B) Cxcl2, and (C) Cxcl1 were measured by real-time PCR. Data are expressed as mean +/− SEM. *Significantly different from vehicle-treated cells. [*Note:* **Significantly different from LPS-treated Kupffer cells at p<0.05].

We next determined the sensitivity of MDMs and Kupffer cells to LPS. Treatment of Kupffer cells with LPS increased Tnf-α, Cxcl1, and Cxcl2 mRNA levels by 15.9, 1.6, and 2.3-fold respectively (Figure 4A–B). Treatment of MDMs with LPS increased Tnf-α, Cxcl1, and Cxcl2 by 102.9, 3.2, and 8.2-fold respectively (Figure 4A–C).

### Generation of chimeric mice

**Figure 5:**
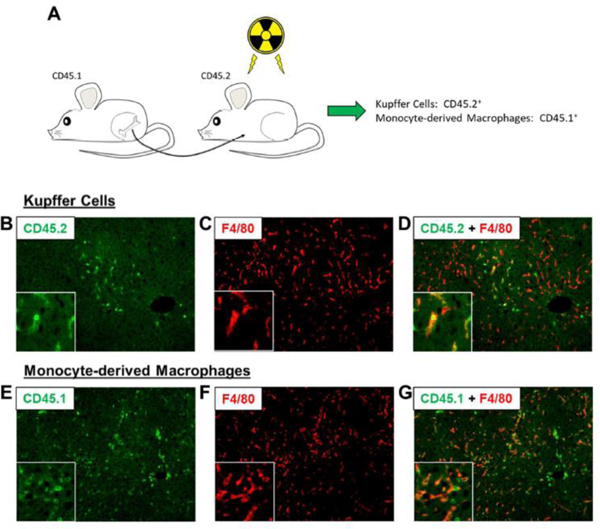
(A) C57BL/6 (i.e., CD45.2) mice were subjected to whole body irradiation followed by transplantation with bone marrow from CD45.1 mice. (B-D) Immunohistochemistry was used to detect CD45.2 (i.e., Kupffer cells) and F4/80. (E-G) Immunohistochemistry was used to detect CD45.1 (i.e., MDMs) and F4/80.

**Figure 6:**
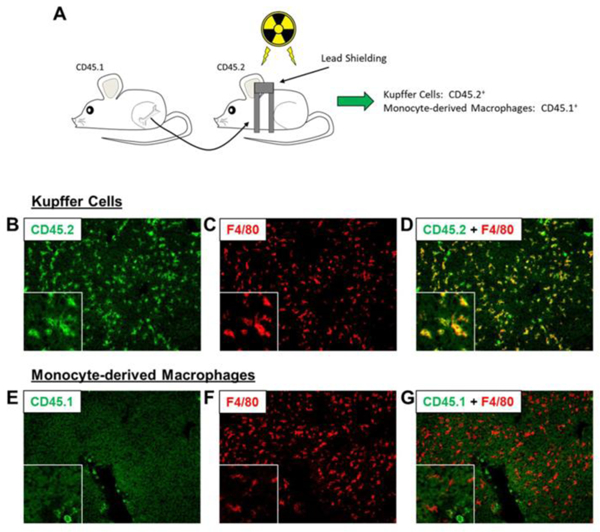
(A) C57BL/6 (i.e., CD45.2) mice were subjected to partial body irradiation followed by transplantation with bone marrow from CD45.1 mice. (B-D) Immunohistochemistry was used to detect CD45.2 (i.e., Kupffer cells) and F4/80. (E-G) Immunohistochemistry was used to detect CD45.1 (i.e., MDMs) and F4/80.

We next used bone marrow transplantation to generate chimeric mice. To accomplish this, bone marrow was isolated from mice expressing the CD45.1 allele and transplanted into lethally irradiated mice expressing the CD45.2 allele (Figure 5A). Because Kupffer cells are of embryonic origin, they remain CD45.2^+^ after transplant, whereas MDMs, which arise from hematopoietic stem cells in the bone marrow, will be CD45.1^+^. After bone marrow transplant, we first used immunofluorescence staining to confirm that Kupffer cells were F4/80^+^CD45.2^+^ whereas MDMs were F4/80^-^CD45.1^+^. As anticipated, CD45.2 positive cells (green) colocalized with F4/80 (red) (Figure 5B–5D). Surprisingly, though, there was also substantial colocalization between CD45.1 (green) and F4/80 (red), indicating that many F4/80^+^ Kupffer cells had arose from hematopoietic stem cells in the bone marrow (Figure 5E–5G). It is possible that whole body irradiation produced extensive Kupffer cell toxicity that required MDMs, recruited from bone marrow, to fully recover. To prevent Kupffer cell toxicity, we shielded the liver with lead prior to lethal irradiation (Figure 6A). As shown in Figure 6B–D, after bone marrow transplant, all F4/80^+^ cells were CD45.2^+^ (Figure 6B–6D) whereas all CD45.1^+^ cells were F4/80^-^ (Figure 6E–G). This indicated that Kupffer cells were CD45.2^+^ whereas MDMs were CD45.1^+^.

### Isolation of Kupffer cells and MDMs from chimeric mice and treatment with LPS

**Figure 7:**
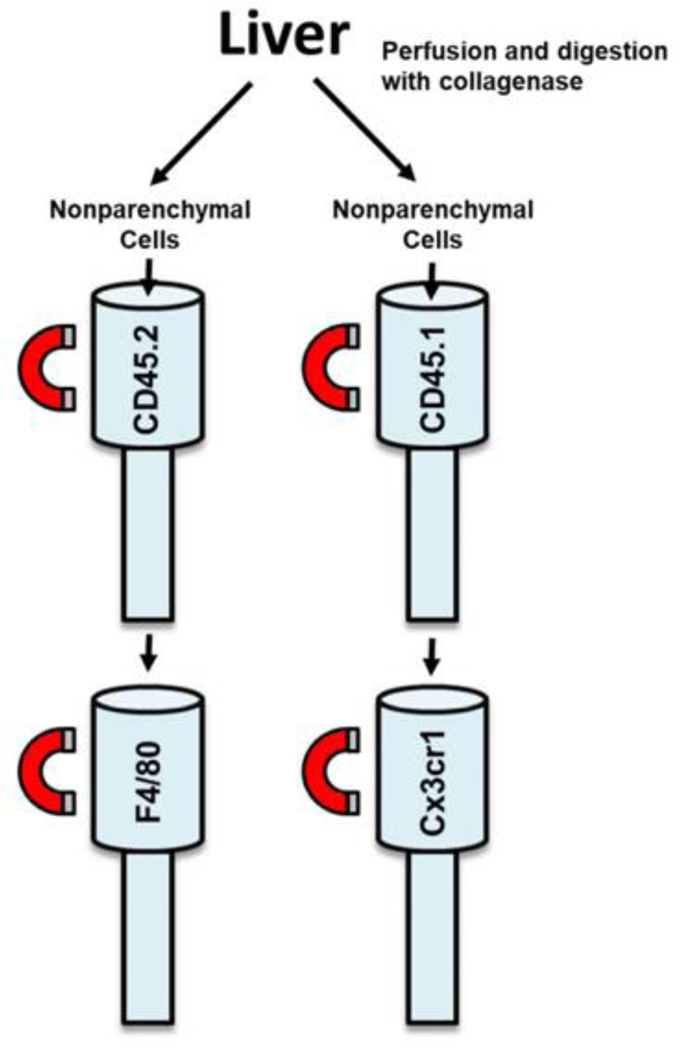
Livers from bone marrow transplanted mice were perfused and digested with collagenase. Nonparenchymal cells were separated from hepatocytes by centrifugation. Kupffer cells were purified from the nonparenchymal cell fraction via immunomagnetic bead separation using antibodies against CD45.2 and F4/80. MDMs were purified from the nonparenchymal cell fraction via immunomagnetic bead separation using antibodies against CD45.1 and Cx3cr1.

**Figure 8:**
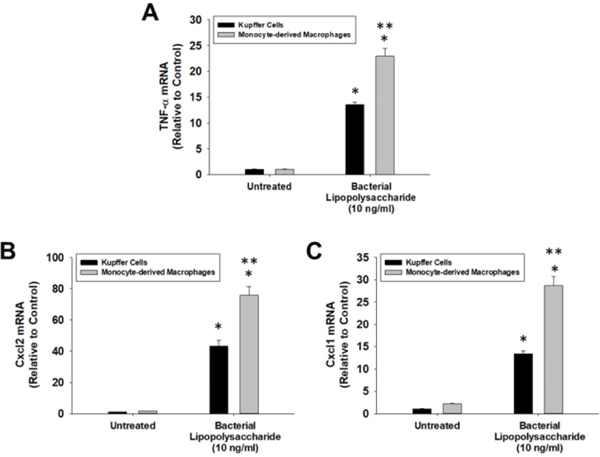
Kupffer cells and MDMs were isolated from the livers of mice subjected to bone marrow transplantation as illustrated in Figure 6 and 7. The cells were treated with LPS or vehicle for 3 hours. mRNA levels of (A) TNF-α, (B) Cxcl2, and (C) Cxcl1 were measured by real-time PCR. Data are expressed as mean +/− SEM. *Significantly different from vehicle-treated cells. **Significantly different from LPS-treated Kupffer cells at p<0.05.

Kupffer cells and MDMs were isolated from bone marrow transplanted mice with lead shielding. Magnetic beads labeled with CD45.2 and F4/80 were used to isolate Kupffer cells, whereas magnetic beads labeled with CD45.1 and CX3CR1 were used to isolate MDMs (Figure 7). Treatment of Kupffer cells, isolated in this manner, with LPS increased Tnf-α, Cxcll, and Cxcl2 mRNA levels by 13.6, 13.4, and 43.1-fold respectively (Figure 8). Treatment of MDMs with LPS increased Tnf-α, Cxcl1, and Cxcl2 by 22.9, 28.7, and 75.9-fold respectively (Figure 8).

## Discussion

Recent studies demonstrated that hepatic MDMs and Kupffer cells possess distinct transcriptomic profiles [8,9,11]. This suggested that functional differences may exist between these two hepatic macrophage populations. In support of this, our studies revealed that proinflammatory cytokines were upregulated to a greater extent in MDMs when compared to Kupffer cells. In addition, basal levels of Tnf-α were higher in MDMs. These results indicate that MDMs are skewed towards an M1-like proinflammatory phenotype and may produce a greater inflammatory response when exposed to bacteria or potentially other pathogens. It is possible that these phenotypic differences between MDMs and Kupffer cells stem from differences in their anatomical location within the liver. MDMs reside proximal to the Glisson’s capsule putting them in close proximity to the peritoneal cavity [9]. Interestingly, studies have demonstrated that MDMs extend dendrites into the peritoneal cavity where they are able to capture bacteria. Based upon these findings, it was proposed that MDMs are important for preventing the dissemination of bacteria from the peritoneal cavity into the liver [8]. Pathogens, such as those originating from the gut, can migrate through the wall of the peritoneal cavity and rapidly spread into the systemic circulation [15]. Bacterial infection in the peritoneal cavity occurs infrequently but is often fatal [16]. It is possible that the ability of MDMs to produce a potent inflammatory response and capture peritoneal bacteria is responsible for the rare occurrence of peritoneal infections.

By contrast to MDMs, Kupffer cells are frequently exposed to pathogens or other immunoreactive materials entering the liver from the portal circulation. For example, studies have shown that LPS concentrations are higher in the portal circulation when compared to the systemic circulation [17,18]. Kupffer cells have a high capacity to phagocytose pathogens, however, our studies indicate that this may not stimulate a potent inflammatory response. It is possible that Kupffer cells produce a diminished inflammatory response to prevent development of chronic inflammation elicited by the persistent exposure to gut-derived pathogens. While our studies indicate that MDMs and Kupffer cells are differentially sensitive to LPS, a limitation of the approach used for this study is that the cells were isolated from the liver prior to treatment with LPS. It is possible that this may have impacted the sensitivity of the cells to LPS. Additional studies aimed at investigating this difference in vivo are needed to validate these in vitro observations.

While the mechanistic basis for the differential sensitivity of MDMs and Kupffer cells to LPS is not known, it is possible that it may result from differential expression of components of the LPS signaling pathway. Surprisingly, though, comparison of the genetic profiles of MDMs and KCs showed that components of the LPS signaling pathway, such as Tlr4, were actually enriched in Kupffer cells [8,9,19]. Another possible explanation for the decreased sensitivity of Kupffer cells to pathogens, is that Kupffer cells are exposed to greater levels of anti-inflammatory mediators. In support of this, Kupffer cells were shown to express higher levels of the anti-inflammatory cytokine, interleukin-10 (Il-10) [9]. IL-10 is a potent anti-inflammatory cytokine, that inhibits secretion of proinflammatory cytokines and other inflammatory mediators [20,21]. Increased expression of IL-10 by Kupffer cells may therefore function to inhibit LPS-induced upregulation of proinflammatory cytokines. Further studies are needed, however, to test this possibility.

In our studies, mice that received whole body irradiation (no shielding of the liver) followed by bone marrow transplantation, resulted in livers populated with macrophages that co- stained for CD45.1 and F4/80, as well as macrophages that co-stained for CD45.2 and F4/80. This indicated that a population of mature Kupffer cells originated from bone marrow-derived monocytes. At the time of completion of our studies, this contradicted what had been reported in the literature, which stated that Kupffer cells only arise from local proliferation of mature Kupffer cells in the liver. Studies since, however, have reported that when a high percentage of liver resident Kupffer cells are depleted, bone marrow-derived monocytes can fill the empty niche and fully differentiate into self-renewing, fully differentiated Kupffer cells exhibiting the same transcriptional profile as yolk sac-derived Kupffer cells [7,9]. This indicates that the Kupffer cell phenotype is generated by cues received locally in the liver and that MDMs can become Kupffer cells if a proper niche is available.

In conclusion, our studies demonstrate that Kupffer cells and MDMs are differentially sensitive to pathogens with MDMs demonstrating enhanced induction of proinflammatory cytokines after exposure to LPS. This differential sensitivity may have evolved to prevent peritoneal bacterial infections and chronic hepatic inflammation. Furthermore, if the specific components of the local niche that control the differential sensitivity of MDMs and Kupffer cells to LPS could be identified, they could potentially be manipulated by drugs to either enhance clearance of bacteria or to reduce inflammation during chronic hepatic inflammation.
